# A minimal 3D model of mosquito flight behaviour around the human baited bed net

**DOI:** 10.1186/s12936-020-03546-5

**Published:** 2021-01-07

**Authors:** Jeff Jones, Gregory P D Murray, Philip J McCall

**Affiliations:** Department of Vector Biology, Liverpool School of Tropical Medicine, Pembroke Place, L3 5QA Liverpool, UK

**Keywords:** Mosquito, Malaria, Vector control, Host-seeking, Bed net, Behaviour, Computational model, *In silico*, tracking, Insecticide, Repellent, Anopheles, Attractants

## Abstract

**Background:**

Advances in digitized video-tracking and behavioural analysis have enabled accurate recording and quantification of mosquito flight and host-seeking behaviours, facilitating development of individual (agent) based models at much finer spatial scales than previously possible.

**Methods:**

Quantified behavioural parameters were used to create a novel virtual testing model, capable of accurately simulating indoor flight behaviour by a virtual population of host-seeking mosquitoes as they interact with and respond to simulated stimuli from a human-occupied bed net. The model is described, including base mosquito behaviour, state transitions, environmental representation and host stimulus representation.

**Results:**

In the absence of a bed net and human host bait, flight distribution of the model population was relatively uniform throughout the arena. Introducing an unbaited untreated bed net induced a change in distribution with an increase in landing events on the net surface, predominantly on the sides of the net. Adding the presence of a simulated human bait dramatically impacted flight distribution patterns, exploratory foraging and, the number and distribution of landing positions on the net, which were determined largely by the orientation of the human within. The model replicates experimental results with free-flying living mosquitoes at human-occupied bed nets, where contact occurs predominantly on the top surface of the net. This accuracy is important as it quantifies exposure to the lethal insecticide residues that may be unique to the net roof (or theoretically any other surface). Number of net contacts and height of contacts decreased with increasing attractant dispersal noise.

**Conclusions:**

Results generated by the model are an accurate representation of actual mosquito behaviour recorded at and around a human-occupied bed net in untreated and insecticide-treated nets. This fine-grained model is highly flexible and has significant potential for *in silico* screening of novel bed net designs, potentially reducing time and cost and accelerating the deployment of new and more effective tools for protecting against malaria in sub-Saharan Africa.

## Background

Whether to combat insecticide resistance, exploit new knowledge of vector biology, increase community acceptance or to accommodate changes in abiotic conditions, mosquito vector control methods are under constant pressure to improve. In sub-Saharan Africa, where 90% of malaria occurs, long-lasting insecticidal nets (LLINs) have been the main driving force in the reduction of malaria cases [1, 2], but widespread and growing insecticide resistance to the pyrethroid insecticides used on nets has stalled, and threatens to reverse, recent advances [3–6]. Novel insecticide compounds used either alone or in combination with existing pyrethroids, provide one solution [7, 8] but this raises significant questions regarding the optimal placement of compounds on the net walls or roof, both for maximizing safety and efficacy and minimizing cost. A detailed understanding of the spatio-temporal nature of mosquito responses to various insecticidal treatment(s) on the bed net interface is critical to balance these competing requirements for the numerous combinations possible.

The development and eventual implementation of a new bed net is an expensive and time-consuming process and the pipeline from early phase screening of chemicals with potential through the range of laboratory and field tests needed to generate the evidence required by regulatory authorities before the commodity finally reaches the affected communities, can take up to ten years.

The first studies on the behaviour of mosquitoes at the bed net interface demonstrated that significantly greater numbers of mosquitoes landed on the bed net roof [9, 10]. Later technological developments in imaging and computing enabled tracking of entire flight paths [11, 12], and 3D reconstructions of arrival patterns [13, 14] revealed complex but consistent behaviours, ultimately revealing how insecticide treatments on bed nets affect the behaviour of malaria vector mosquitoes. The growing body of data arising from those studies not only builds the evidence base required to accelerate the development process, but it also provides an excellent foundation for developing models of host-seeking behaviour with potential to validate experimentally the new tools at earlier stages. One such model is presented here: a fine-grained agent-based approach for modelling how indoor insecticide treatments deployed as residues on bed nets affect the behaviour and survival of mosquitoes.

To successfully find, select and feed on a human host, mosquitoes process input from multiple sensory modalities with the relative importance of particular cues differing at different stages of the approach. These include isolated carbon dioxide (CO_2_) concentration, olfactory [15–17], visual [18, 19], auditory [20, 21] and tactile sensory cues [22], or combinations thereof [23].

The interaction between host-seeking mosquitoes and the human host begins long before the mosquito enters the house but the quantity of sensory information is likely to be at its most intense in a room around a human-occupied insecticidal bed net. This is a unique context for a mosquito behaviour model as most modelling approaches to disease transmission by insect vectors either set the scale at a much higher level, emphasizing interactions at village scale (and above) [24, 25], or at the opposite end of the scale, modelling individual mosquitoes in experimentally controlled settings such as wind tunnels and olfactometers [26].

Until there is a more thorough understanding of the mechanisms guiding mosquito host location processes, the development of accurate fine-grained models of insect flight behaviour and host interactions will be restricted. Instead, agent-based modelling provides an alternative approach because, as noted in [27], it can represent stochasticity and heterogeneity at fine-grained scales and generate emergent behaviours not explicitly encoded into the model. Previous agent-based approaches to mosquito flight behaviour have included a 2D model of insect flight to study orientation and tracking of odour plumes [28], and a 2D model of mosquito interactions with insecticidal bed nets which was used to assess community scale protection [29].

The unresolved complexity of the roles and relative importance of different sensory stimuli were circumvented by developing a minimally simple agent-based model of 3D mosquito flight behaviour. The model is stimulus-agnostic and uses a single generic ‘attractant’ signal emanating and dispersing from a host. The model was used to study mosquito flight behaviour and landing distribution patterns on unoccupied and human-occupied bed nets. The eventual aim is to employ the model as a virtual experimental tool for accelerated exploration and evaluation of novel bed net treatments or designs. Hence, model parameters were validated with the results from previous experimental work on mosquito behaviour at the bed net interface.

## Materials and methods

The Indoor Vector Control Testing System (InVeCTS) model creates a virtual environment in which to assess mosquito populations’ interactions with their host and environment. It uses an agent-based approach with fine-grained spatial representation in which a mosquito population can interact with a human host emanating the hypothetical spatially distributed attractant stimulus over time. Mosquito flight occurs in real time and all mosquito flight paths and interactions with the environment are recorded for subsequent analysis.

### Environment and mosquito representation

**Fig. 1:**
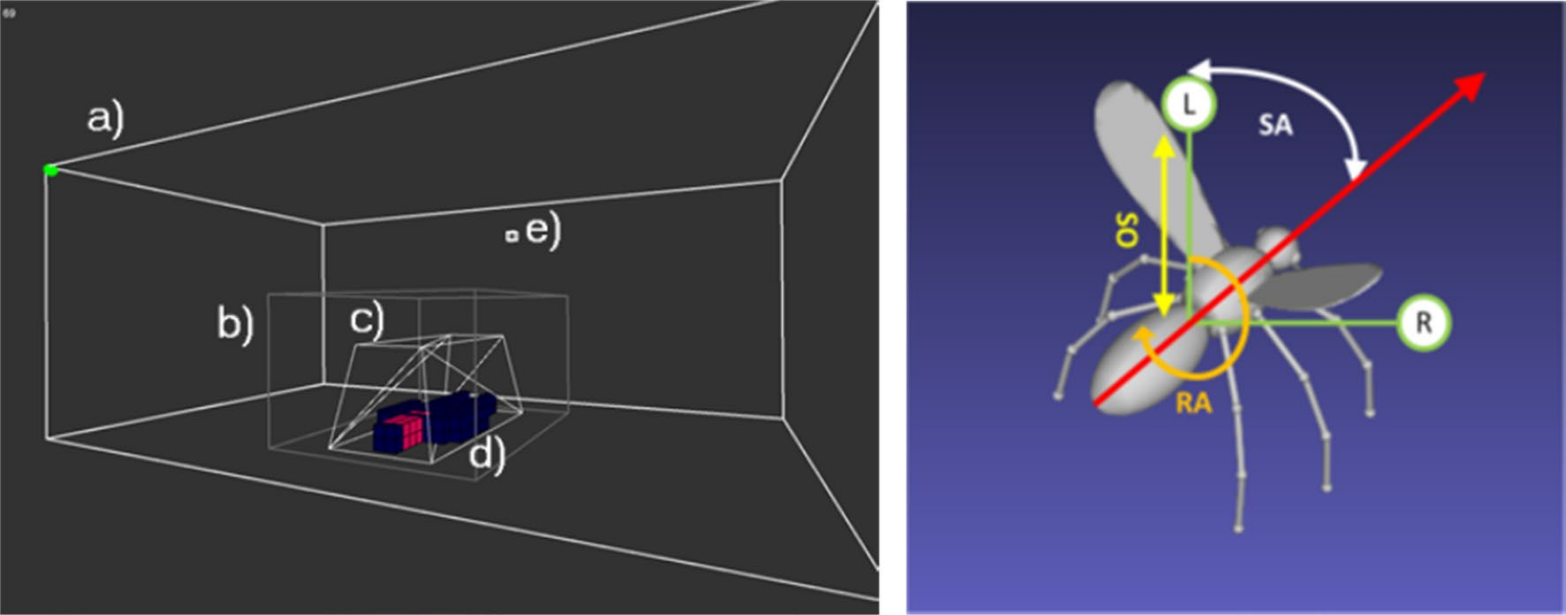
Arena and mosquito representation in the InVeCTS model. Left: Mosquito flight environment. The environment consists of the main insectary arena comprising **a** a cuboidal recording volume corresponding to that used in [11] **b**, a bed net **c** containing a human host **d**. Mosquito release location is indicated in **e**. Right: Schematic representation of virtual mosquito agent with its current directional vector (long arrow), its offset Left and Right sensors (L,R), Sensor Offset distance (SO), Sensor Angle (SA) and Rotation Angle (RA) parameters

A population of 25 mobile virtual mosquitoes is created. The flight tracking system data used to explore the parameter space of the model mosquitoes was generated from experiments with adult female *Anopheles gambiae sensu stricto (s.s.)*, generally considered the most important vector of all, using the “Kisumu” strain, a widely used colony-reared insecticide susceptible strain. These individuals fly in a continuous 3D space representation inside a discrete spatial arena, representing an insectary whose dimensions directly correspond to an experimental insectary at Liverpool School of Tropical Medicine (LSTM) (5.6 m long × 3.6 m wide × 2.3 m high). This virtual insectary can contain a bed net and human host, as shown in Fig. 1.

The population is introduced at the release site (Fig. 1e) and begins exploration of the arena. A hypothetical attractant plume is projected with the size and shape of a human host of approximately 180 cm in height. To represent and store the attractant profile the arena is divided into a $56\times 36\times 23$ cubic lattice of $10~cm^3$ cells. The host bait profile is configurable to represent hosts of different sizes (Fig. 1d) and the cells making up the bait profile are colour-coded to indicate regions where greater concentrations of attractant are emanated. As previously stated, a wide range of environmental cues (for example, CO$_2$, skin odours, sound, vision, temperature) are known to influence mosquito behaviour but their relative contribution and sequential importance in host location is still uncertain [30–32]. To circumvent this uncertainty, and to simplify the model, a generic representation of a single spatial attractant emanating and diffusing from the host was used. This is a simplifying assumption that the multi-modal nature of the attractant profile can be represented in this manner. A simple cellular automata-based dispersion mechanism was used in order to enable real-time dispersal of the attractant profile.

### Projection and dispersal of human bait attractant

At each model time step, attractant is projected at bait profile locations and isotropic dispersion is implemented in parallel for all cells in the 3D lattice volume by distributing the attractant between the current cell and the 26 nearest neighbour cells. Vertical dispersion (a very coarse approximation of convection) is implemented for all cells by dispersing a fraction of each cell to the cell immediately above the current cell in the arena. Such a simple scheme was used for two reasons. Firstly, this is because of the uncertainty of the relative importance of different environmental cues, and secondly, it enables the visualization of the dispersal in real-time which is not yet possible with more complex representations of environmental dispersion.

Real-time projection, diffusion and convection of bait profile attractant in the lattice is parameterized by independent weight parameters for diffusion ($W_d$) and convection ($W_c$). At every time step *t* a generic chemo-attractant, weighted by the bait profile map (a simple representation of a human form resting within the bed net, see Fig. 1,d), is projected into the 3D attractant lattice at locations directly corresponding to the bait map profile shape. The bait profile map values are weighted to account for regions projecting higher levels of attractant, for example, from the mouth.

#### Attractant plume diffusion

At every model time step *t*, volumetric diffusion of attractant is approximated by the following cellular automaton-based method.

For each cell in the attractant 3D lattice (axes x,y,z): $C_{xyz}^{(t+1)}$ = (($C_{xyz}^t$ + local neighbourhood attractant values (radius 1))/27) * $W_d$

To approximate non-uniform dispersal of attractant (for example, turbulent air currents) and their effects on host-seeking behaviour each cell can be modified by adding a value randomly sampled from a Gaussian distribution with mean 0 and standard deviation 1. Perturbation by Non-uniform dispersal can be increased by multiplying this noise value by a scaling parameter $D_s$.

#### Attractant plume convection

Directly after the diffusion step volumetric convection upwards is approximated by copying of vertically stacked horizontal ‘sheets’ of cells containing attractant values to cells in the sheets above (Insectary ceiling is set to $Y=0$, layers below are $Y+1$ etc). Projection is weighted by convection weight parameter $W_c$.

For each cell in the attractant lattice (axes *x*, *y*, *z*): $C_{xyz}^{(t+1)}$ = ($C_{x(y+1)z}^t$) * $W_c$

**Fig. 2:**
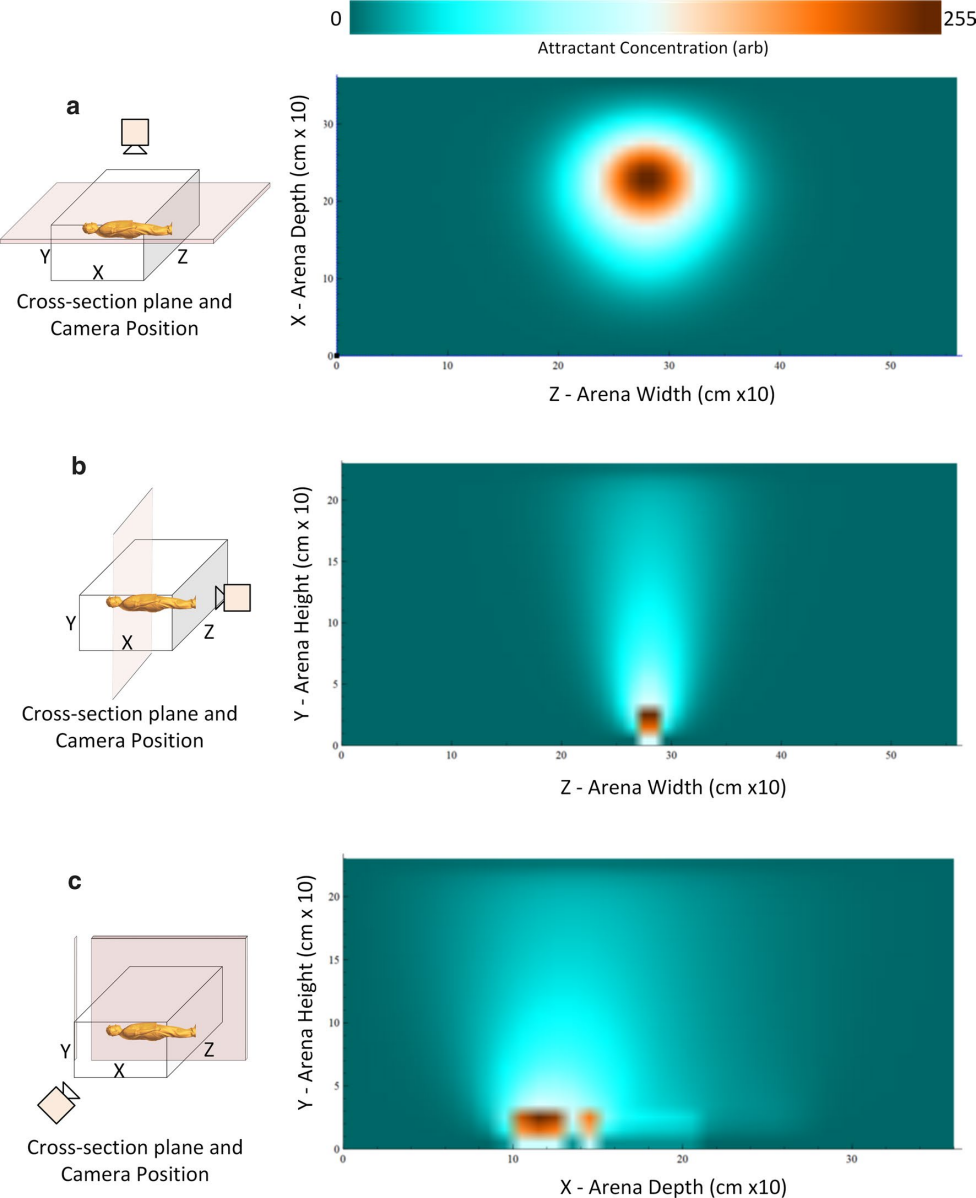
Visualisation of attractant plume within the arena. Left side shows orientation of cross-sectional slice through attractant field within the arena and camera position. Right side shows attractant concentration scaled individually for each image to the look-up table (top). **a** Gradient concentration of attractant plume (looking from above insectary, slice taken from coronal plane, 0.7m above floor) **b** Gradient concentration of attractant plume (looking along axial plane of human profile). Section is taken at the head region of the human profile. **c** Gradient concentration of attractant plume (looking at side-on human profile). Section is taken as a saggital slice along the middle of the human profile

A visualization of the effect of attractant plume dispersal is shown in Fig. 2. To reiterate, this is a simple approximation of attractant dispersion. However, it is sufficient to generate a stable 3D attractant field and avoids emphasizing any one particular (potentially erroneous) stimulus type or sensory modality over others.

### Mosquito sensory-motor Algorithm

After the diffusion and convection stage each mosquito samples the attractant value in the insectary from two offset sensors (sensor offset distance may be adjusted with the parameter **SO**. Sensor angle may be adjusted with the parameter **SA**. If the sensor values are different the agent orients itself locally in space by rotating about its own axis towards the strongest value, by the amount in degrees given by the Rotation Angle (**RA**) parameter. Additionally, the agent orientation is subject to random modification by the **pCD** (probability of Change Direction) parameter. If a randomly sampled value from the uniform distribution is $<\mathbf{pCD}$ (default value 0.25), the agent will select a random orientation in 3D space. At each model step each agent moves forwards in its current orientation by a velocity given by the parameter **V** (default value 1 *cm*).

### Mosquito behaviour transition function

Agent behaviour in response to environmental cues is determined by a set of states and the subsequent transition between these states. To avoid a fully deterministic response to population behaviour, transition between states is mediated by probabilistic sampling, implemented by individual parameters, corresponding to a Markov process of state transitions (see Table 1 for all model parameters). A schematic overview of the behavioural transitions can be found in Additional file 1.

**Table 1:** Model parameters. Parameters are divided into groups reflecting the effect on the environment, agent sensory-motor behaviour, and agent behavioural transitions

Parameter type	Name	Default value	Description
Environment	$W_d$	0.7	Diffusion damping factor
	$W_c$	0.3	Convection damping factor
	$D_s$	0	Dispersal noise value
	*tx*	0 (untreated net) 0.1 (treated net)	Net contact toxicity value
Mosquito Flight	SA	45	Sensor angle (deg)
	RA	22	Rotation angle (deg)
	SO	1	Sensor offset distance (cm)
	V	1	Velocity (cm) per model step
Mosquito State	pCD	0.25	Probability of changing direction
	pRest	0.1	Probability of resting on surface
	pLeave	0.05	Probability of leaving surface

The agents are initially in the **PRE-RELEASE** state and their state is updated every model step. A mosquito enters the **FLYING** state when a randomly sampled value from a uniform distribution is $<0.1$. If an agent is at a wall, ceiling, bed net, or floor surface it will rest if a randomly sampled value from a uniform distribution is $<\mathbf{pRest}$ parameter. Non-resting mosquitoes change direction to a random orientation and resting agents stay in the current location until a randomly sampled value from a normal distribution is $< \mathbf{pLeave}$ parameter. It the agent lands on, or is resting on a treated bed net its starting health value (100) is decremented by a toxicity value *tx*. If the health of an agent reaches zero it is removed from the arena.

### Parameter selection and model validation

Model parameters were set to match values obtained by previous image tracking experiments in the same LSTM insectary space [12]. The dimensions of the virtual arena were identical to the insectary, as were positions of the bed net, host, number of mosquitoes, mosquito release location, and experimental run time. Although measurements of mosquito flight speed vary in the literature (and indeed during different mosquito behaviours) a mosquito flight speed of 300 *mm*/*s* was selected to approximate the flight speed recorded experimentally under the same conditions [12].

Varying the *SA* and *RA* parameters affects flight tortuosity which subsequently affects arena occupancy and foraging behaviour. Examples of the effects of varying *RA* parameter can be seen in Additional file 2. After evaluating a range of *SA* and *RA* angle combinations, fixed values were selected, *SA* of 45$^{\circ }$ and *RA* of 22$^{\circ }$ (see Additional files 2 and 3 for the evaluation of sensory parameters and description of tortuosity calculation). This resulted in path tortuosity which approximated those of the image tracking experiments reported in [11]. Limiting assumptions of the model are described in the discussion section.

### Model scheduler and model experiment setup

**Fig. 3:**
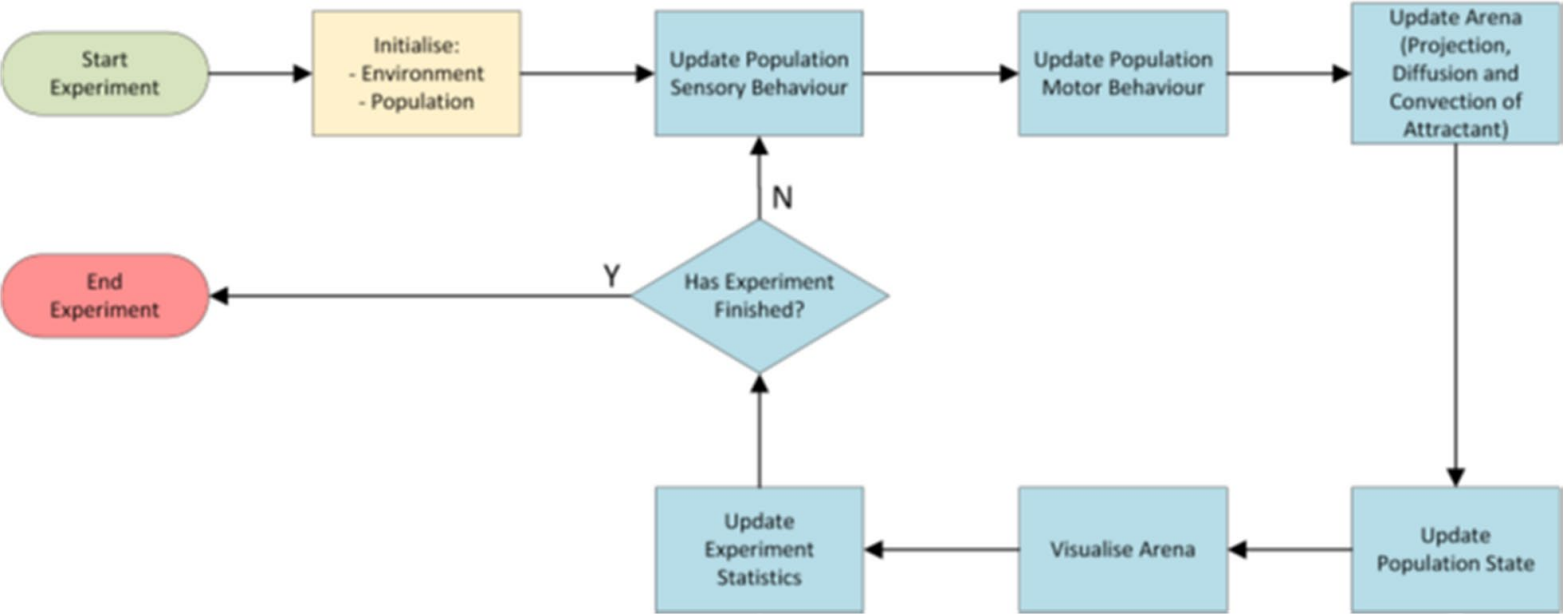
InVECTS model scheduler operation

At the start of an experiment, the system creates the virtual insectary environment and the agent population. The population is placed at the release location and the model begins iterating through its main run-time loop as shown in Fig. 3.

Each model step comprises 1/30 *th* second. The model halts after 1 h, or 108,000 model steps. After the model halts, log files pertaining to virtual mosquito activity (three-dimensional coordinate positions, agent state and agent health), bed net contact locations, and agent presence within spatial regions surrounding the bed net are saved to disk for further analysis.

The user can interact with the model during run-time, visualizing the current state of the model and population flight behaviour in 3D space using a mouse. Specific experimental configurations can be saved and re-loaded for repeated runs.

Five control experiments were performed in the virtual insectary where no bait or no bed net was present, as a baseline for the flight behaviour of the model. Twenty experiments were performed for each of the human bait conditions (no bait, head facing left, and head facing right). The head facing left profile was used assess the effect of noisy attractant dispersal, performing five experiments at each noise parameter value.

## Results

### Spatio-temporal flight activity and foraging behaviour.

**Fig. 4:**
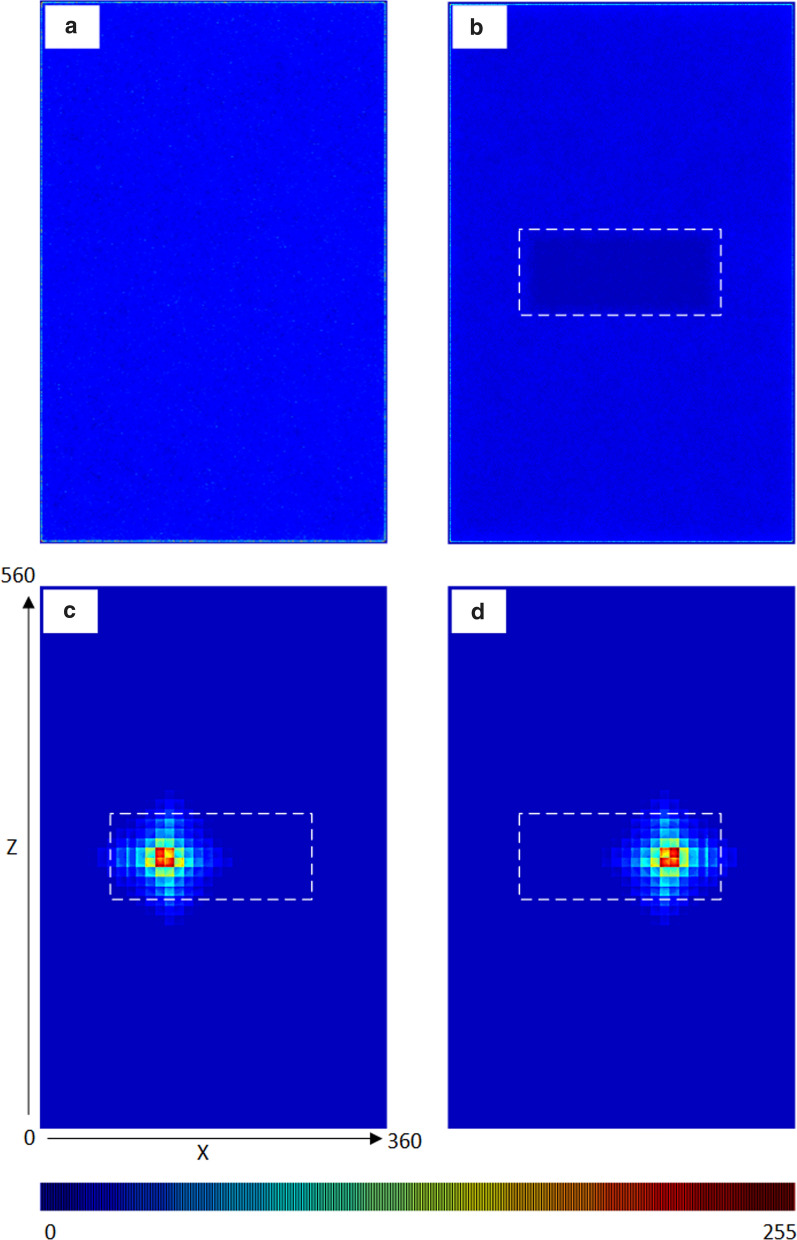
Flight distribution of virtual mosquito population in the simulated insectary. Distribution of activity in the *XZ* plane (i.e. looking from above). Bed net position indicated by dashed rectangle. **a** Insectary containing no bait and no bed net, **b** Insectary with unbaited bed net, **c**, **d** Insectary with head facing left and head facing right human bait. Occupancy at all heights binned to 1 pixel range and scaled to 8-bit look-up table colour values (bottom)

Control experiments, where neither bait nor bed net were present, established baseline flight behaviour and distribution. Occupancy of all regions is plotted in Fig. 4 across the X and Z planes. The heatmap images indicate a top-down summary of occupancy results from above the entire insectary, demonstrating that in the absence of a host or bed net, flight activity was relatively uniform throughout the arena (Fig. 4a).

When an unbaited net is introduced to the arena, the flight distribution pattern in the arena is still relatively uniform except in those regions containing the empty bed net where the mosquito population cannot enter (Fig. 4b). When the stimulus profile of a human shape is placed on the bed, within the net, the flight distribution shows a marked change, with the population showing a strong preference to fly in the regions of the strongest diffusing and convective stimuli, corresponding to the orientation of the human bait profile (Fig. 4c and d).

**Fig. 5:**
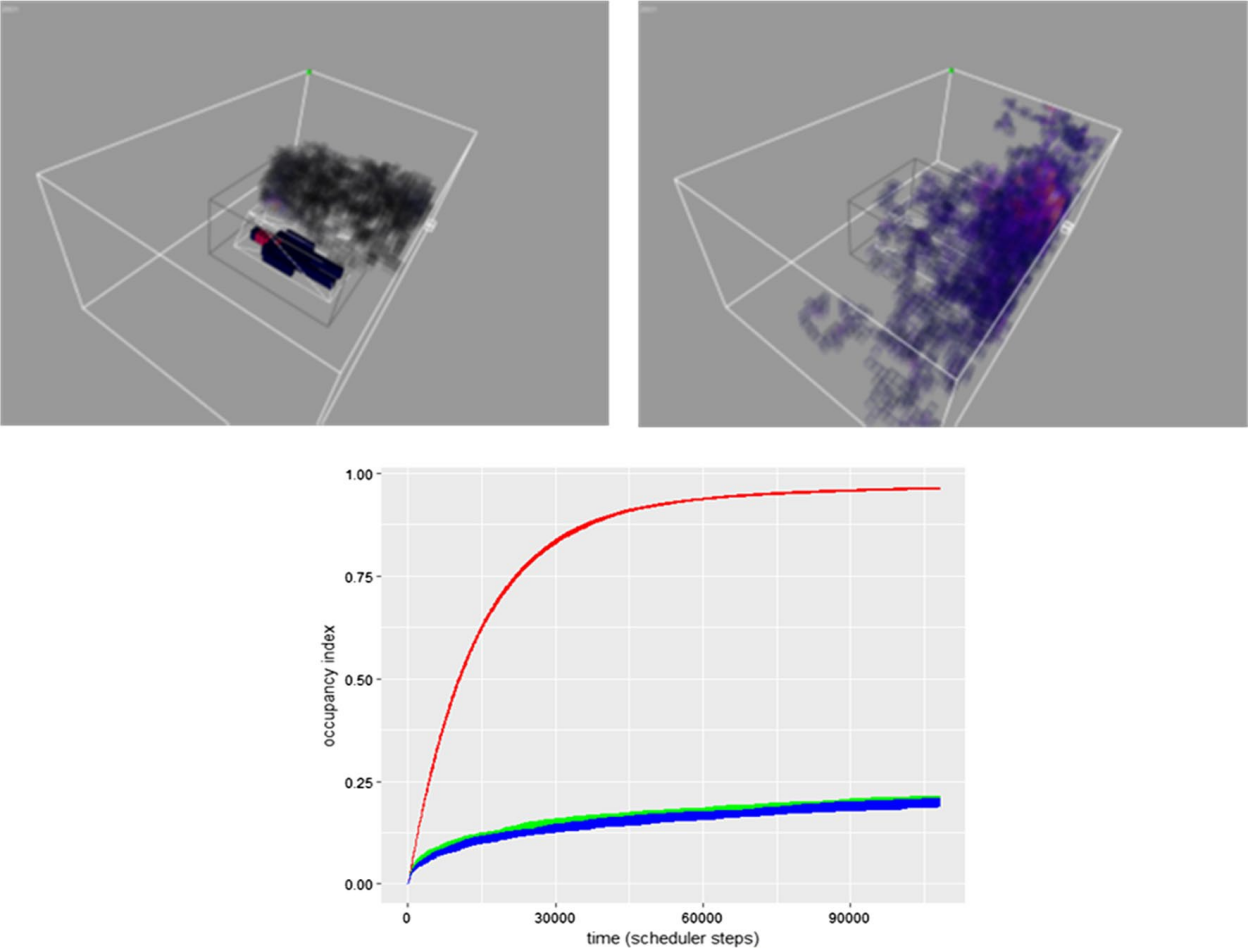
Effect of host presence on foraging and arena occupancy. Top: Snapshot of occupancy in a baited (left) and unbaited (right) arena after 20,000 model steps (approximately 11 min). Partially transparent voxels indicate regions of the virtual insectary which have been occupied. Bottom: Occupancy index (the fraction of the arena explored by the population) for an unbaited bed net (red) compared to baited conditions (green represents head facing left and blue is head facing right)

As in nature [11], foraging in the InVeCTS model population is strongly affected by the presence or absence of a human host. Figure 5 (top) demonstrates spatial foraging at 11 min into a 1-h experiment. In the presence of bait, a narrow field of the arena is explored, whereas the virtual population explores a much larger area of the arena when no host is present. Temporal exploration of arena occupancy over time (the total fraction of the arena explored by the population) confirms that the presence of bait results in less foraging (indicative of behaviour oriented towards a source). When bait is absent the population adopts an exploration strategy, with many casting flights as they seek recognizable host cues. Although it is difficult to compare foraging activity on a per-mosquito basis (due to the high individual variability in flight behaviour) the initial time taken to find and contact the net in each experiment was lower in baited conditions (mean 270 model steps, sd +− 42.23) than in unbaited conditions (307 model steps, sd +− 101). These differing behavioural responses are induced solely by the presence or absence of dispersed attractant stimuli within the arena. Example video recordings of short model runs in unbaited and baited conditions are shown in Additional files 4 and 5 respectively and the mosquito trails indicate differences in foraging behaviour.

### Flight path tortuosity

Flight path tortuosity was calculated using the rolling tortuosity metric described in Additional file 3. To summarize, a sliding window of 50 mosquito movements along the flight path was assessed for all points and only uninterrupted segments of flight paths were used (i.e. those not including landing and resting). Path tortuosity under different bait conditions are shown in Table 2. Tortuosity in unbaited arenas was slightly lower than measured previously in [11], however that study only included information from the recording volume captured by the cameras (where tortuosity is increased by the mosquito responses to the net). The model tortuosity includes the entire insectary space with more free flight space which reduces overall tortuosity. Under baited conditions (which attracts greater mosquito activity at the net) the model tortuosity closely matches experimental values.

**Table 2:** Model vs experimental flight tortuosity

Tortuosity	Unbaited net	Head facing left	Head facing right
Model mean	1.120	1.635	1.640
Model range	1.120–1.120	1.63–1.65	1.63–1.65
Experimental mean [11]	1.31	1.66 (both orientations combined)	
Experimental range	1.16–1.47	1.52 - 1.79	

### Net surface contact distribution

**Fig. 6:**
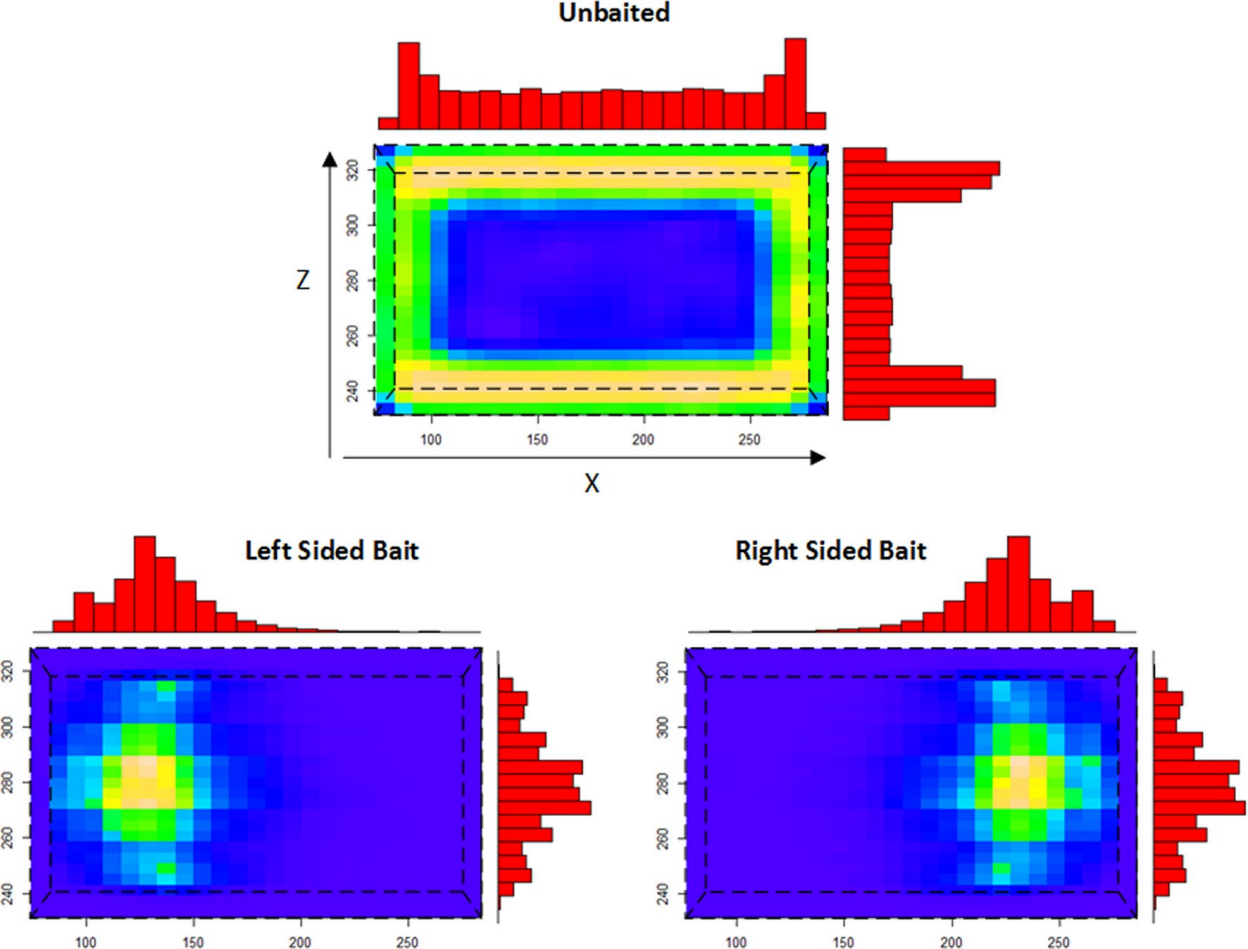
Distribution of bed net contact sites (shown from above, net shape indicated by dashed line). Spatial 2D heat map distribution of occupancy in the bed net XZ plane and 1D frequency distributions. There is relatively little contact at the top of the net in the unbaited condition where most contact occurs at the side net surfaces. In the baited conditions there are a large number of contacts on the top net surface at locations corresponding to the upper torso and head of the host. These contact sites correlate with bait orientation

Net contact locations in the unbaited condition occurred mainly along the sides of the bed net, particularly along the longest axis of the net (Fig. 6, top), with relatively little contact at the top surface of the net.

**Fig. 7:**
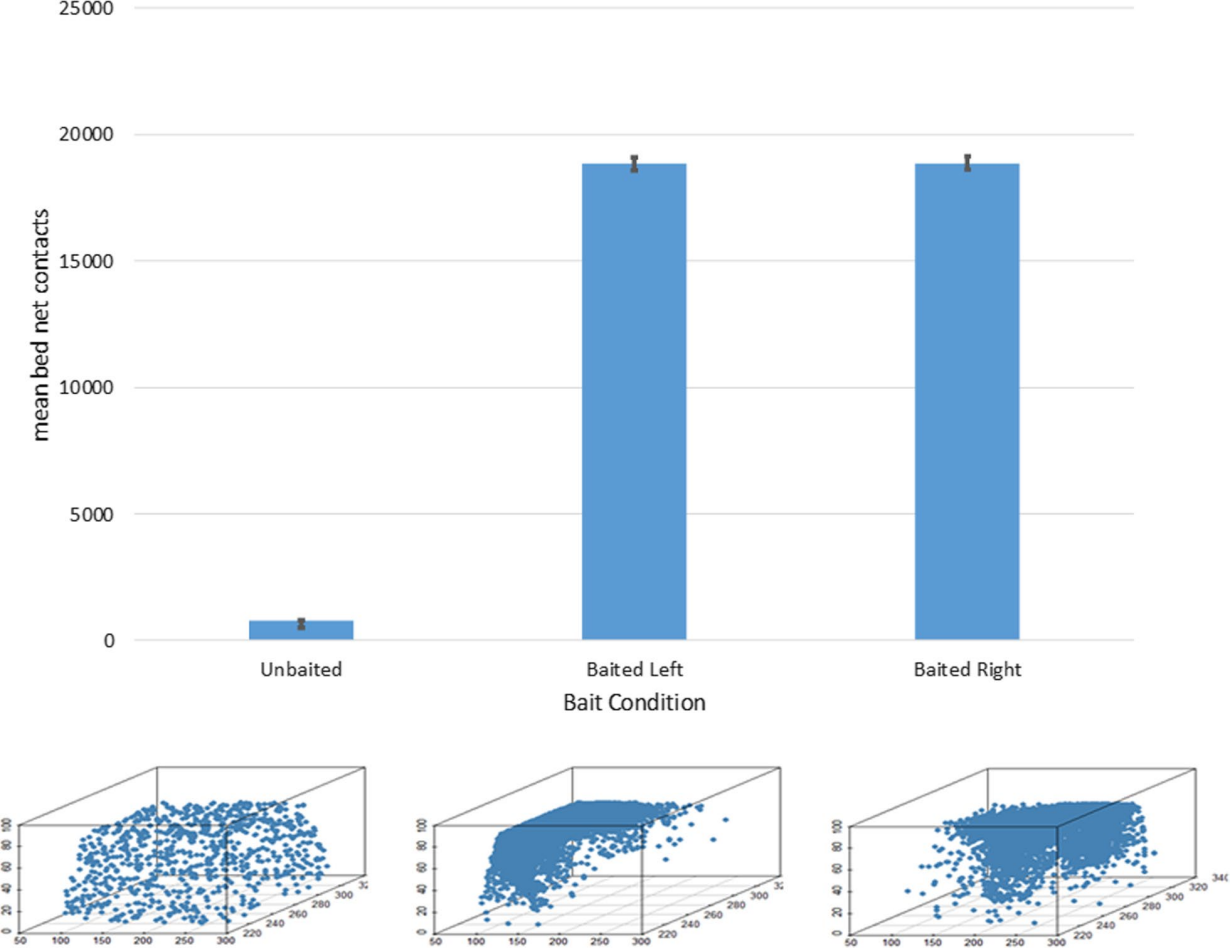
Mosquito net contact distributions. Top: Mean number of unique bed net contact events for each bait condition. Bottom: Example distribution of surface contacts in an experimental run. 3d scatter plot shows circles indicating individual contact sites on all surfaces of the 3d net. Unbaited (left) shows isotropic distribution of contacts. Head facing left (middle) and head facing right (right) bait conditions show distribution of contact sites over the head and torso of the bait stimulus location

Under simulated human bait conditions, contact events showed a predilection for the top surface of the net and the X coordinates of the contact patterns were strongly influenced by the orientation of the human bait beneath (Fig. 6, bottom). An illustration comparing the distribution of net contact numbers and spatial distribution patterns in unbaited and baited experiments is shown in Fig. 7.

The total time spent in contact with the net surface itself is shown in Table 3. The mean contact time for an unbaited net (1.24 m for the model, compared to 2.4 m in the experimental results) is far less than the time spent at the baited net (31.51 m for the model versus 33.1 m in the experimental results). In response to a simulated treated net the model population spent a mean of 13.69 m in contact with the net, compared to 7.3 m in the experimental results. The difference in contact time at the treated net may partly be explained by the fact that the model does not incorporate a representation of internal mosquito energetics which may be responsible for reduced mosquito activity over time [11].

**Table 3:** Model vs experimental Net Contact Time for all contacts

Net Contact Time (m)	Unbaited Net	Baited Net	Treated Net
Model mean	1.24	31.51	13.69
Model range	0.99–1.49	29.45–32.65	13.57–13.77
Experimental mean [11]	2.4	33.1	7.3
Experimental range	2.1–6.8	24.3–41.2	3.9–10.7

Furthermore, the range of contact times over multiple number of experiments in the model is also slightly less than the experimental findings for all bait and treatment conditions. This reflects the high variability of individual mosquitoes and suggests that stochasticity in the model could be increased in future research. This could be achieved by increasing individual virtual mosquito stochasticity (via the probabilistic mosquito state parameters) or by increasing the global stochasticity of the attractant signal via the dispersal noise parameter.

**Fig. 8:**
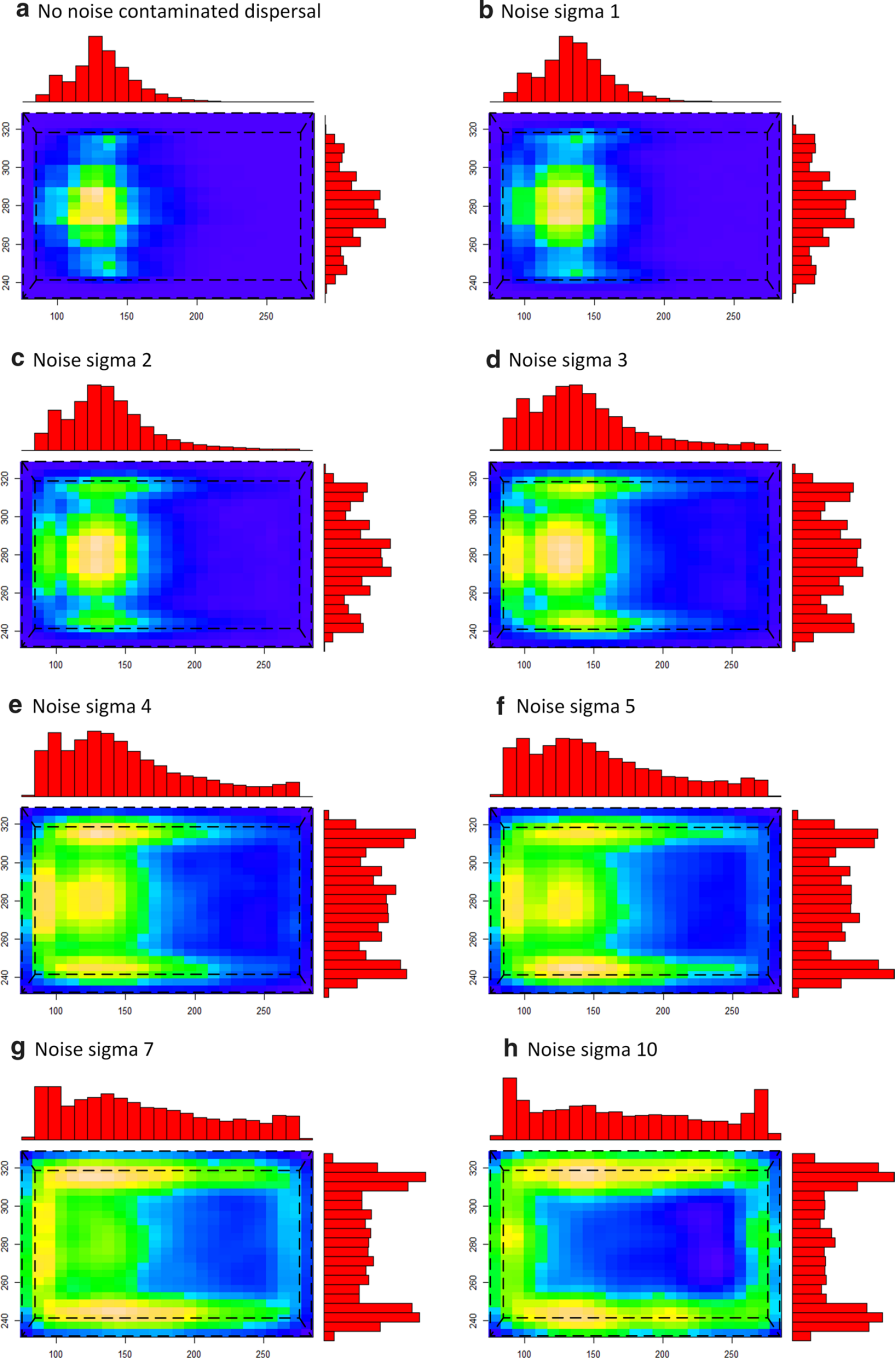
Effect of noise contamination on attractant dispersal and mosquito bed net contact locations. Spatial heat map distribution of mosquito activity in the *XZ* plane (i.e. looking from above, net shape indicated by dashed line) under noisy dispersal conditions with a head facing left host. Gaussian noise sigma multiplier from no noise contamination (**a**) up to sigma 10 (**b**–**f**). Increasing noise spreads mosquito distribution across the top surface of the net and at higher noise levels, increases net contact at the sides of the net

**Fig. 9:**
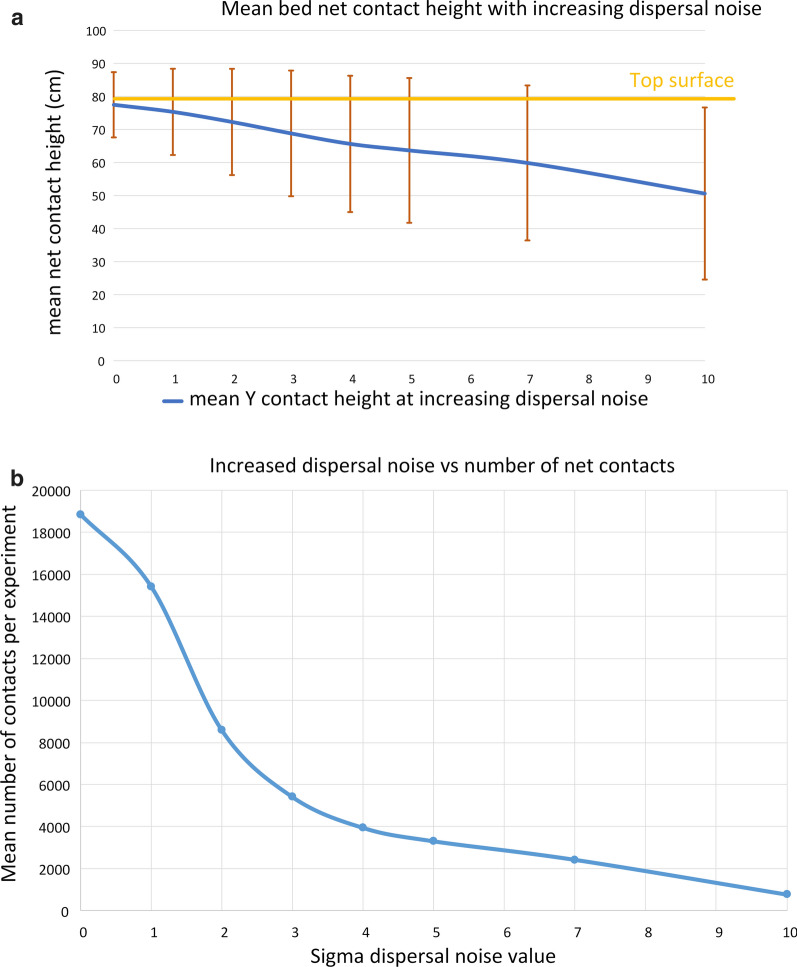
Effect of noise contaminated attractant dispersal on total number of net contacts and contact height. **a** mean net contact height decreases as dispersal noise increases, moving from the top surface (orange) to the sides of the net. **b** The mean number of total net contacts also decreases as dispersal noise increases

It has been suggested that strong air currents may affect host-seeking behaviour and, potentially, subsequent mosquito distribution on bed nets [17]. The effect of noise-contaminated attractant dispersal on virtual mosquito host-seeking and the number and spatial distribution of bed net contact locations was assessed. Figure 8 demonstrates the spatial effects showing, at low noise levels, a wider region of contact on the net surface (Fig. 8a–e). At higher noise levels, the mosquitoes increasingly contact the net at the sides, as opposed to the top surface (Fig. 8f–h). This change of net contact height with increasing noise (Fig. 9a) is accompanied by a reduction in total net contact (Fig. 9b).

### Peripheral bed net region activity

To assess peripheral activity within the regions surrounding the bed net the recording volume was subdivided into polyhedral shapes similar to, but not exactly identical, to the polygonal regions used in an earlier experiment [11]. The reason for the differences is due to the 2D nature of the projection of the image tracking method and the slight difference in net orientation used in [11] (the net was tilted to allow better visualisation of the top surface of the net for the capture cameras). The outer regions of the bed net in the model are divided into 18 regions which allows differentiation between the top surface of the net (12 sub regions), the two end edges of the net (edges corresponding to those near the head and feet) and the two sides of the net (4 regions consisting of arms and legs for both sides of the net). A 3D visualization of the peripheral regions is shown in Additional file 6. Occupancy of the regions is recorded during experimental runs. Each time a mosquito is located within a particular region, a counter for that region is incremented. The total mean region distribution data for control and baited conditions is summarized in Additional file 7.

**Fig. 10:**
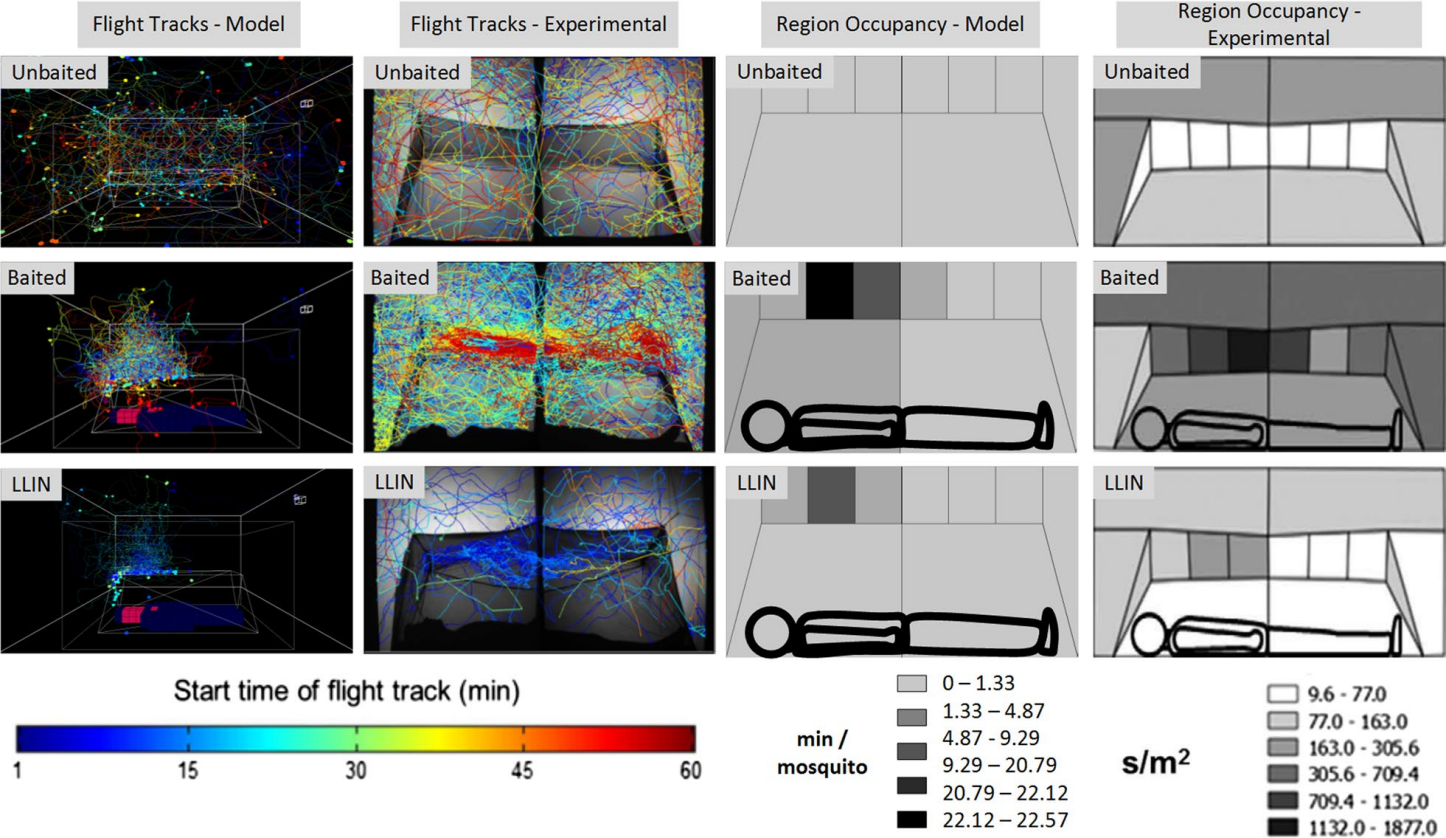
Spatio-temporal flight tracks and peri-bed net regional occupancy. Simulation (Cols 1 & 3) and experimental (Col 2 & 4) data. Track colour indicates time (scale for flight tracks indicated below Cols 1 & 2). Region colour indicates mean occupancy time indicated below Cols 3 & 4. Conditions shown are for an unbaited net (top row), an untreated net (middle row) and LLIN treated net (bottom row). Model tracks show a single mosquito for clarity and circular regions denote landing sites. Experimental images reproduced courtesy of [11]

A visual summary of flight tracks and peripheral region occupancy of the model output compared to experimental data in response to unbaited, untreated and insecticide-treated nets is shown in Fig. 10 and the data in Additional file 7. In the absence of a human host, there is slightly greater activity in regions to the side and ends of the bed net than in regions just above the net. This is partly due to the elongated nature of the virtual insectary, but also because the two sides of the net present a larger surface area than the roof of the net. When a host is present in the net, however, the activity profile changes markedly. The attractant stimuli diffusing from the human host profile strongly attracts the mosquito population to the top regions surrounding the net. The presence of a baited net also affects flight activity in the peripheral regions in total, resulting in approximately 8 times as much peri-bed net activity compared to an unbaited net. A baited net coated with insecticide ($tx= 0.1$ ‘dose’ accumulation per contact, decrementing an initial health value of 100) exhibits the same strong attraction to the top surface of the net. Repeated contact, however, diminished the health of the mosquitoes until the population was entirely killed in a mean time of 36.34 m (s.d. 2.56 m). This corresponds closely with the findings from experimental tracking which found that activity after 30 m was negligible on LLIN treated nets [11].

## Discussion

The human home is exploited by numerous parasitic arthropods, including vectors of malaria, dengue, yellow fever, lymphatic filariasis, leishmaniasis and Chagas disease. In Africa, most cases of malaria are transmitted indoors, despite the wide range of behavioural preferences shown by those Anopheles spp. that are vectors [33, 34]. In turn, humans have exploited this behaviour using a number of methods to target them at various locations inside the home. Of the methods used to date, insecticide-treated bed nets (ITNs/ LLINs) have been shown to be highly effective [1], and they remain the main intervention in malaria prevention in Africa. As a first stage in developing models that capture vector behaviour from house entry to exit, incorporating spatial movement and resting site preferences, this agent-based model of mosquito flight and host-seeking dynamics was developed, based on the actual conditions and their impacts on vector behaviour measured previously [11].

This is the first fine-grained model to simulate mosquito flight and distribution patterns in 3D space from quantification of actual tracked flight behaviours recorded in [11] and similar findings from earlier studies [9, 10]. Notably here, the patterns of net contact and flight distribution in the peri-bed net region were accurately reproduced by the model. Specifically, the differences in number of net contacts in no-host and host-present conditions, the preference for the top surface of the bed net (which is of critical importance relating to the design of next-generation bed nets and with respect to damaged nets [35]), the orientation of net contacts depending on host orientation, and patterns of flight activity in regions surrounding the bed net.

It is the combined effect on this behaviour of the attractants emanating from the host within the net and the potentially repellent or irritant properties of the insecticide treatment on the net, that determine whether or not a mosquito makes sufficient net contact to acquire a lethal dose. That this is accurately represented in the model is shown by Fig. 10. Without a host acting as attractant, activity around the bed net was relatively uniform (Fig. 10, top row), reflecting the shape of the arena and positioning of the bed net. When a human host was present, the behaviour of the mosquito population changed in response to different stimulus conditions, aggregating preferentially on the roof of the bed net (Fig. 10, middle row). The foraging behaviour of the virtual population measured by an occupancy metric based on the fraction of the arena explored, shifted from an ‘exploration’ behaviour (wide spatial casting throughout the arena) to an ‘exploitation’ behaviour (a narrower focused exploration as the population oriented to an source of attractants). This difference in behaviour was entirely provoked by the diffusion of the attractant plume from a host. With an untreated net this foraging and attempted penetration of the net persists over the entire hour whereas LLIN treated nets, in experimental and simulation conditions, dramatically reduced the activity of the mosquito population (Fig. 10, bottom row).

Turbulent dispersal of the attractant field was approximated using a noise parameter which added Gaussian noise during the diffusion method. Increasing dispersal noise resulted in fewer net contacts, a wider distribution pattern and, at high noise values, a change in contact distribution from the top surface of the net to the sides of the net.

### Limitations of the model

These results demonstrate that a fine-grained modelling approach has utility as an *in silico* method of performing virtual mosquito bio-assay experiments to explore 3D indoor flight behaviour. As with all modelling approaches, limiting assumptions have been made. Here, the most significant are associated with host attraction: visual stimuli are not represented and the multiple sensory modalities known to have a major role as attractant stimuli were greatly simplified. The latter, the spatially dispersed stimuli − CO$_2$, host odour (from the body and exhaled breath) and body temperature are known to be of major importance in host location and conveniently were suitable for grouping. Although other sensory modalities also contribute [31, 36], the interactions are complex and not yet fully elucidated, whereas the mosquito behaviours seen at insecticidal bed nets can be explained in terms of the these grouped stimuli alone. This simplification was also driven by the need for real-time simulation performance. Alternate methods of attractant dispersion and sampling, for example, computed by Computational Fluid Dynamics (CFD) methods, are a future possibility, although this would likely bias the model to particular mechanisms of stimulus dispersion.

The model mosquitoes do not incorporate any approximation of energy expenditure and therefore do not display a tail-off in flight activity over time, as previously reported in [11]. The tracking data used for the basis of the model was based on the behaviour of a single mosquito species (*An. gambiae s.s.*). However, published data from tracking experiments shows that, in terms of spatial activity, the behaviour of *Anopheles arabiensis* [37] and *Culex quinquefasciatus* [12] at human-baited ITNs is remarkably similar to that of *An. gambiae*.

The relative simplicity of the mosquito behavioural stimulus-response transitions was chosen to attempt to reduce the wide range of potential parametric influences on model behaviour, a known issue with agent-based approaches. Nevertheless, despite these limiting assumptions, the model has demonstrated that it is able to reproduce the broad findings of [9, 10], most notably that the distribution of mosquito landing sites occurs predominantly on the top surface of the net and is affected by disturbances in dispersion by shifting this distribution to lower regions of the net. Furthermore, the model also accurately reproduces experimental tracking findings of [11], exhibiting similar flight tortuosity, bed net contact time, peri-bed net distribution and activity decay in response to simulated insecticide-treated bed nets.

### Application of the model and scope for further work

By making adjustments to the model parameters, the model can accommodate far more variation than described here. Examples would include: additional vector mosquitoes with different arrival patterns at the bed net or different host species preferences (certain resistant strains of *An. gambiae* mosquitoes, for example, exhibit different activity levels during flight tracking than susceptible strains); the influence of a second host (human or other) in the room; effects of the new insecticide treatments on the bed net (level of pre-contact repellency or post-contact irritation), whether applied to all surfaces equally or to the roof alone; combining the insecticidal bed net with residual insecticide sprayed on the wall.

Ongoing experimental evaluation of the so-called next generation bed nets (bi-treated nets with pyrethroid and non-pyrethroid insecticides) will greatly expand the model’s power to predict how new net treatments perform, and determine whether they are best deployed alone or in combination.

Recently, an expanded version of the model was used to evaluate a range of novel bed net designs, and rapidly identified the best of seven candidate(s) [38]. The rapid process means the new design will be evaluated in initial trials in sub-Saharan Africa in early 2021. This research demonstrates that simple models of mosquito flight around bed nets can assist in enabling rapid assessment of novel vector control tools.

## Supplementary Information

**Additional file 1.** Figure transition function. Schematic flowchart of mosquito behaviour transition function.

**Additional file 2.** Model Parameter Selection. Virtual mosquito turn angle (RA parameter) affects foraging and occupancy behaviour and flight path tortuosity. Top Figure illustrating how RA parameter affect arena occupancy and flight path tortuosity. Bottom Figure showing individual effects of SA and RA parameters on sensing the attractant plume. A general description of model parameter selection follows.

**Additional file 3.** Chart flight tortuosity. hart showing effect on flight path tortuosity of the RA parameter. A description of how the flight path tortuosity metric is calculated.

**Additional file 4.** S1 Video unbaited condition. Simulated flight behaviour in unbaited condition. Video recording of simulated mosquito flight behaviour in response to an unbaited bed net. Population of 25 mosquitoes.

**Additional file 5.** S2 Video baited condition. Simulated flight behaviour in baited condition. Video recording of simulated mosquito flight behaviour in response to a human occupied bed net. Population of 25 mosquitoes.

**Additional file 6.** Fig. regions subdivision. Subdivision of peri-bed net regions. Subdivision of space peripheral to the bed net into 18 regions comprised of 3D polyhedra covering the top surface (coded as 0-11 white sub-regions, 0-5 (top strip), 6-11 (bottom strip)), short ends (12 and 13, (red and green respectively)) and side regions (14-17, blue (top) and magenta (bottom) respectively).

**Additional file 7.** Table regional occupancy. Peri-bed net region occupancy. Mean time per mosquito (s) occupying each region surrounding the bed net for each condition. Regions and their subregions indicated on left side. Mean counts and Standard Deviation per condition. Summary table indicating total activity time per mosquito (m) for net surface contacts and occupancy within peri-bed net regions.

## Data Availability

Results and data materials available from authors on request.
