# Supplementary figures and images for: A minimal 3D model of mosquito flight behaviour around the human baited bed net
Source: Malar J. 2021 Jan 7;20:24. doi: 10.1186/s12936-020-03546-5 (PMC7792054; doi:10.1186/s12936-020-03546-5)

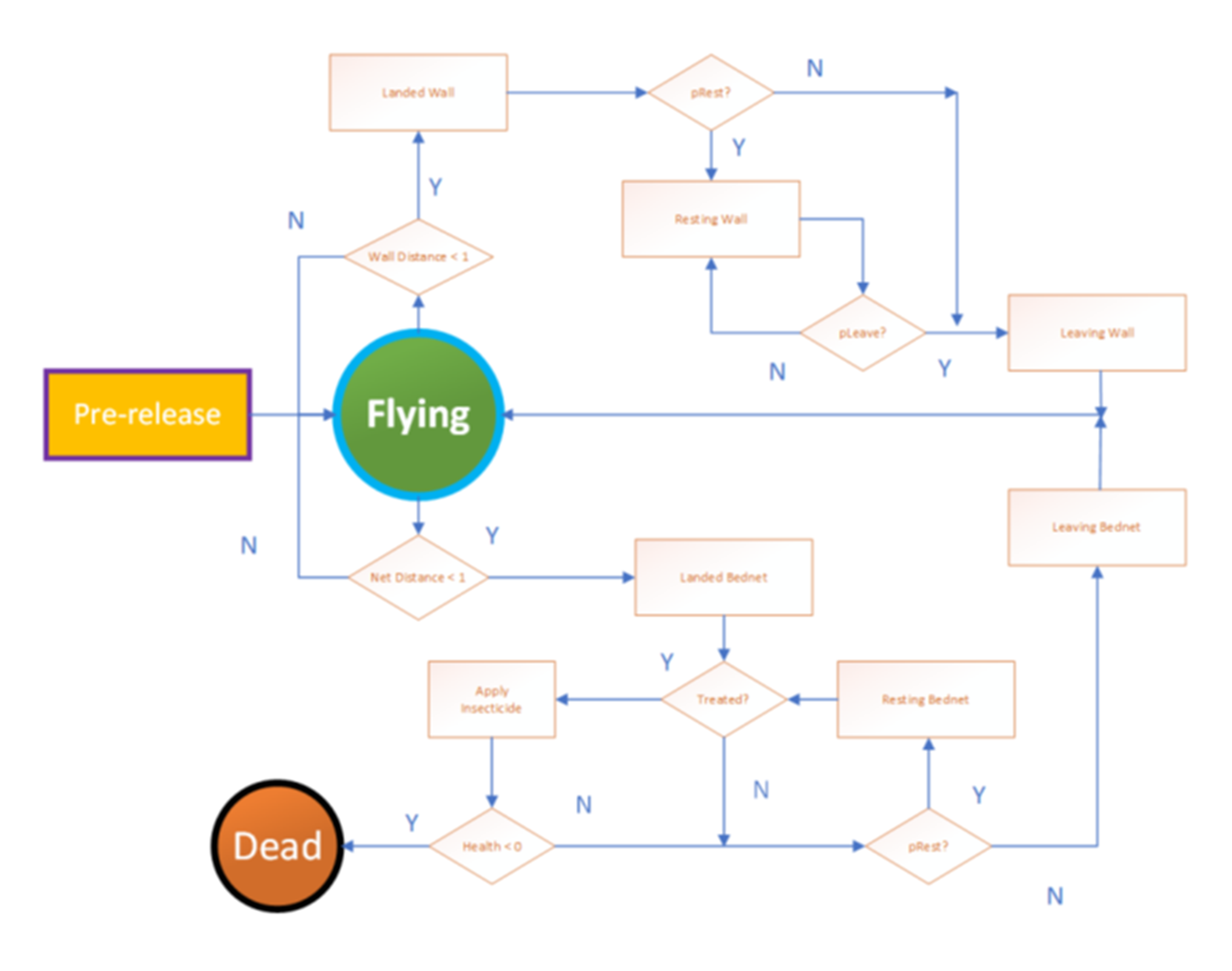

Supplement: Supplementary file 1 — Additional file 1. Figure transition function. Schematic flowchart of mosquito behaviour transition function. [file 12936_2020_3546_MOESM1_ESM.png]

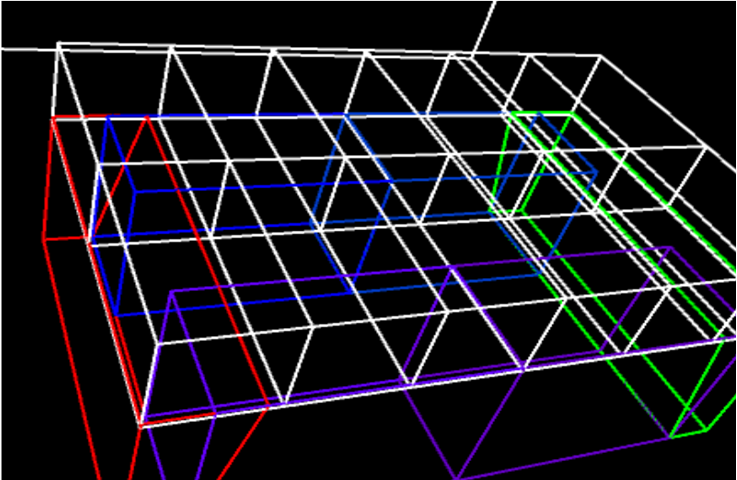

Supplement: Supplementary file 6 — Additional file 6. Fig. regions subdivision. Subdivision of peri-bed net regions. Subdivision of space peripheral to the bed net into 18 regions comprised of 3D polyhedra covering the top surface (coded as 0-11 white sub-regions, 0-5 (top strip), 6-11 (bottom strip)), short ends (12 and 13, (red and green respectively)) and side regions (14-17, blue (top) and magenta (bottom) respectively). [file 12936_2020_3546_MOESM6_ESM.png]
