# Supplementary material for: A minimal 3D model of mosquito flight behaviour around the human baited bed net
Source: Malar J. 2021 Jan 7;20:24. doi: 10.1186/s12936-020-03546-5 (PMC7792054; doi:10.1186/s12936-020-03546-5)
Supplement: Supplementary file 2 — Additional file 2. Model Parameter Selection. Virtual mosquito turn angle (RA parameter) affects foraging and occupancy behaviour and flight path tortuosity. Top Figure illustrating how RA parameter affect arena occupancy and flight path tortuosity. Bottom Figure showing individual effects of SA and RA parameters on sensing the attractant plume. A general description of model parameter selection follows. [file 12936_2020_3546_MOESM2_ESM.pdf]

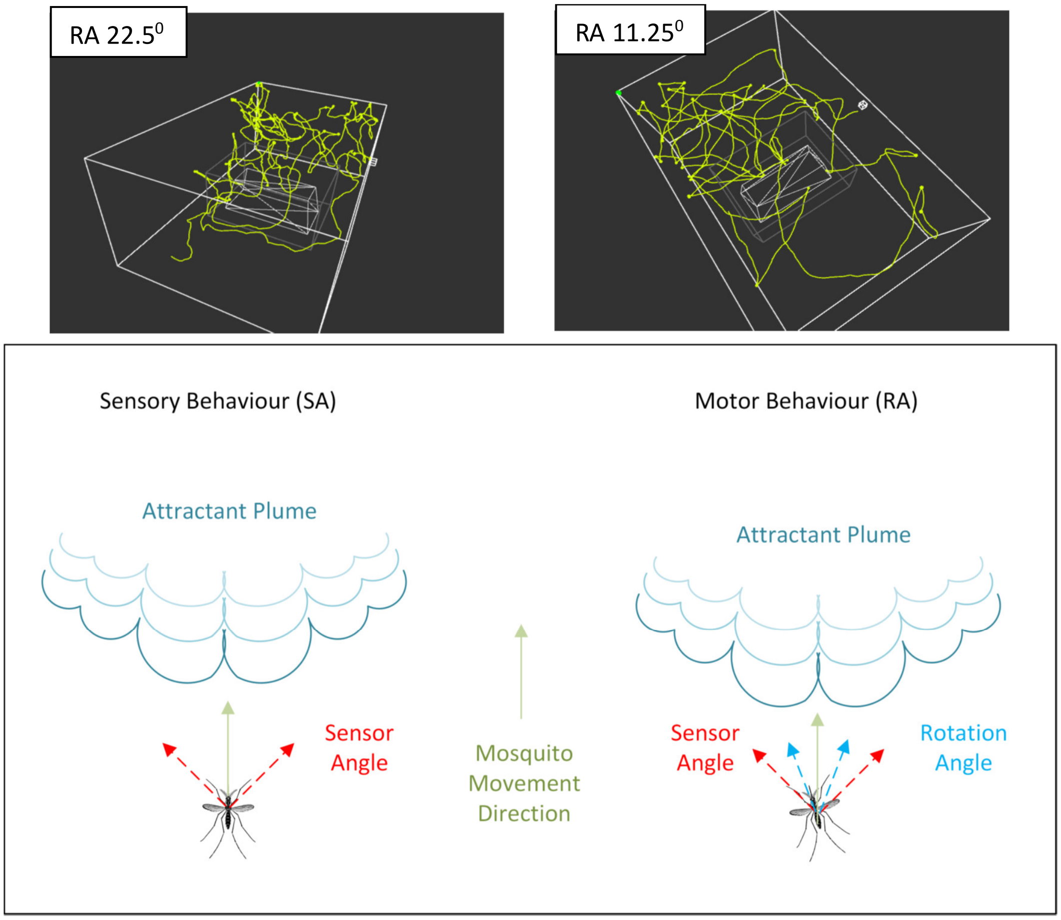


**S6 Fig.**

Virtual mosquito Sensor angle (SA parameter) and turn angle (RA parameter) affects foraging and occupancy behavior and flight path tortuosity. Top: Examples of 3D flight paths from a single agent in unbaited arena with SA at $45^0$ and turn angles of $90^0$ (top left), $67.5^0$ (top right), $22.5^0$ (middle left) and $11.25^0$ (middle right).

Bottom: Illustration of effect of SA and RA parameters. Left: A sensor angle of $45^0$ ensures that the mosquito can distinguish between different spatial concentrations within the oncoming attractant plume. Right: The RA parameter affects exploration within the attractant field. Large RA values result in greater local foraging within the current region. Small angles result in less foraging in the current region. A rotation angle of $22^0$ was found to keep the mosquito within the attractant plume signal path yet still allow sufficient exploration to track the attractant signal during flight.

Model Parameter Selection:

The majority of the environmental parameters were selected from flight tracking experiments of [11], including arena dimensions, net and host location, mosquito release site and experiment run times. Mosquito flight parameters were both derived from the literature (for example mosquito flight speed from tracking experiments) and empirically derived during model development. Mosquito sensory and movement parameters were empirically derived because, although there are detailed recordings of mosquito flight in the literature, these are a mixture of 2D and 3D recordings and under a range of different environmental conditions (restricted within olflactometers, within relatively small cuboidal volumes, within large swarms and with artificially induced discrete inputs of $CO_2$ stimuli). As there are no existing 3D models of mosquito flight behaviour we based flight parameters based on a forward-biased movement with SO value of 1 as an approximation of the mosquito size. Velocity of 1 cm/s, combined with model scheduler update rate of 30 steps results in 300 mm/s flight speed, corresponding to that typically observed during tracking experiments. SA and RA parameters are tightly coupled and a SA of 45 degrees was quickly established during model development as the most suitable value which kept forward mosquito movement facing the attractant plume during forward movement. Variations in the RA parameter have a more subtle effect. In real mosquito flight the rotation angle during flight in response to a changing local attractant levels is likely to be affected by a vast number of environmental and neural cues. However, a kinematic model incorporating these dynamics is obviously not possible.

We therefore used a fixed rotation value of 22 degrees which we found most closely recorded flight tortuosity in the tracking data and yielded the observed differing behavioural responses (in terms of number of net contacts and peri-bed net region occupancy) with the presence or absence of host attractant respectively. The stochastic parameters were used to reproduce the large variation seen in mosquito tracking data. The **pCD** parameter (random probability of change in direction) ameliorates some of the consequences of the fixed RA parameter. The **pRest** and **pLeave** parameters were used to allow flexibility when selecting different mosquito strains (and potentially species) as we have noted that some resistant strains show less activity overall despite having similar flight behaviours (unpublished data).
