# Supplementary material for: A minimal 3D model of mosquito flight behaviour around the human baited bed net
Source: Malar J. 2021 Jan 7;20:24. doi: 10.1186/s12936-020-03546-5 (PMC7792054; doi:10.1186/s12936-020-03546-5)
Supplement: Supplementary file 3 — Additional file 3. Chart flight tortuosity. hart showing effect on flight path tortuosity of the RA parameter. A description of how the flight path tortuosity metric is calculated. [file 12936_2020_3546_MOESM3_ESM.pdf]

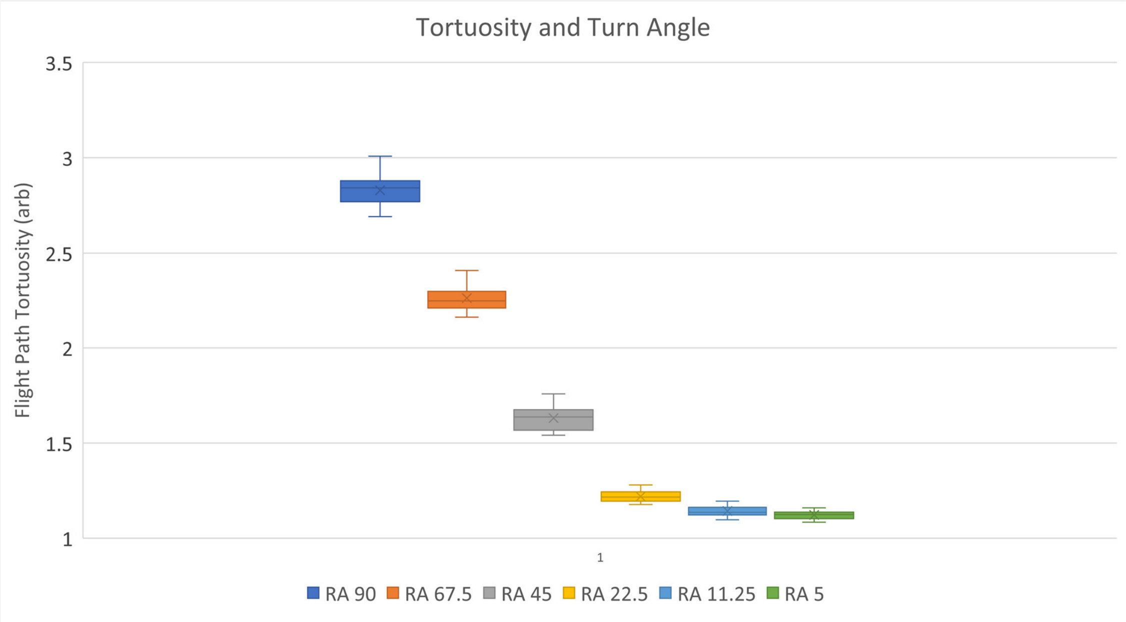

S7 Chart.

Plot of changing flight path tortuosity metric at different turn angles. Flight path tortuosity is computed per mosquito, per time step as a comparison between 50 contiguous flight movements in a moving window. The window covers 50 scheduler steps of *uninterrupted* movement throughout the entire 1hr experiment and path comparison is by comparing mosquito positional vector at each of these 50 steps against the Euclidean straight line between start point t_0 and end point t_{50}. A value of 1 would indicate a perfectly straight track between the points. Values greater than 1 indicate higher path tortuosity. Higher path tortuosity corresponds to increased foraging within a particular spatial region. Lower path tortuosity corresponds to decreased foraging within a particular spatial region.
