# Supplementary material for: A minimal 3D model of mosquito flight behaviour around the human baited bed net
Source: Malar J. 2021 Jan 7;20:24. doi: 10.1186/s12936-020-03546-5 (PMC7792054; doi:10.1186/s12936-020-03546-5)
Supplement: Supplementary file 7 — Additional file 7. Table regional occupancy. Peri-bed net region occupancy. Mean time per mosquito (s) occupying each region surrounding the bed net for each condition. Regions and their subregions indicated on left side. Mean counts and Standard Deviation per condition. Summary table indicating total activity time per mosquito (m) for net surface contacts and occupancy within peri-bed net regions. [file 12936_2020_3546_MOESM7_ESM.pdf]

S5 Peri-bed net occupancy Tables.

Peri-bed net region occupancy. Mean time (s) per mosquito occupying each spatial region surrounding the bed net for each condition. Regions and their sub regions indicated on left side. Mean counts and Standard Deviation per condition.

Region	Sub Region	No Net or Bait 5 runs (s)	SD	Unbaited Net 20 runs (s)	SD	Left Sided Bait 20 runs (s)	SD	Right Sided Bait 20 runs (s)	SD	LLIN Treated Net 0.1/contact (s)	SD
Top	R0	10.49	0.82	10.57	0.99	95.46	2.51	0.58	0.22	42.83	3.71
	R1	10.22	0.55	10.34	0.91	709.85	9.81	6.01	1.18	316.30	18.10
	R2	10.73	0.44	10.41	0.97	347.15	6.63	46.78	2.64	153.24	11.50
	R3	10.76	0.31	10.51	0.98	49.93	2.57	348.43	6.57	22.78	2.82
	R4	10.21	0.75	10.38	1.25	7.09	1.17	689.45	10.39	3.44	0.92
	R5	10.05	1.12	10.57	0.91	0.92	0.32	96.33	2.96	0.50	0.32
	R6	9.81	0.45	10.31	0.88	93.98	3.24	0.71	0.31	41.98	3.46
	R7	10.36	0.36	10.49	0.65	642.50	9.46	5.42	0.85	287.58	17.47
	R8	10.05	0.76	10.52	0.74	272.47	6.82	39.08	2.07	119.71	9.72
	R9	9.89	0.40	10.27	0.85	36.69	2.10	269.06	5.62	16.69	2.11
	R10	10.70	0.97	10.17	0.93	5.11	0.82	664.58	8.01	2.49	0.96
	R11	10.82	0.98	10.58	0.85	0.81	0.24	95.84	2.90	0.38	0.23
Ends	R12	29.67	1.09	29.03	3.12	94.99	6.14	0.72	0.47	41.53	5.47
	R13	30.29	1.29	29.54	2.03	0.77	0.53	96.15	5.88	0.40	0.42
Sides	R14	27.36	0.91	26.64	2.90	81.24	4.89	3.97	0.76	36.04	4.34
	R15	27.55	1.79	26.43	2.75	3.75	0.84	81.75	3.89	1.54	0.76
	R16	26.17	1.34	25.98	2.70	72.54	4.62	3.38	0.88	33.73	5.49
	R17	25.79	1.45	25.94	1.96	3.15	0.71	73.54	4.90	1.51	0.71
**Total (s)**	**All**	**290.91**		**2518.40**		**2521.78**		**2520.09**		**1122.68**	

Summary of the mean time spent (minutes per mosquito) in contact with the bed net itself and within all regions surrounding the bed net. In the unbaited condition there is only 4.81 minutes spent in regions surrounding the net, compared to 42 minutes in the baited condition. The effect of a treated bed net reduces the regional occupancy to 18.71 minutes as the mosquito population is killed off.

	Total Net Contact Time (m per mosquito)	Total time spent in Regions Surrounding Bed net (m per mosquito)
No Net or Bait Present	N/A	4.85
Unbaited Net	0.05	4.81
Untreated Net	1.26	42.00
LLIN Treated Net	0.55	18.71
